# Persistent Occurrence of *Cryptosporidium hominis* and *Giardia duodenalis* Subtypes in a Welfare Institute

**DOI:** 10.3389/fmicb.2018.02830

**Published:** 2018-11-20

**Authors:** Yuanfei Wang, Na Li, Yaqiong Guo, Lin Wang, Rui Wang, Yaoyu Feng, Lihua Xiao

**Affiliations:** ^1^State Key Laboratory of Bioreactor Engineering, School of Resources and Environmental Engineering, East China University of Science and Technology, Shanghai, China; ^2^Key Laboratory of Zoonosis of Ministry of Agriculture, College of Veterinary Medicine, South China Agricultural University, Guangzhou, China

**Keywords:** *Cryptosporidium*, *Giardia duodenalis*, *Enterocytozoon bieneusi*, children, welfare institute

## Abstract

Few data are available on the transmission dynamics of intestinal protozoa in children in welfare institutes. In this study, fecal specimens were collected from 396 children in a welfare institute in Shanghai, China during December 2011 (207 specimens), June 2012 (78 specimens), and September 2013 (111 specimens), and examined for *Cryptosporidium* spp., *Giardia duodenalis*, and *Enterocytozoon bieneusi* by PCR analysis of the small subunit rRNA, triosephosphate isomerase, and internal transcribed spacer genes, respectively. The *Cryptosporidium hominis* and *G. duodenalis* assemblage A identified were further subtyped by multilocus sequence typing. Altogether, *Cryptosporidium* was detected in 39 (9.8%) children, with infection rates of 11.6% (24/207), 9.0% (7/78), and 7.2% (8/111) in December 2011, June 2012, and September 2013, respectively. Infection rates were higher in children of 0–12 months (20.4% compared to 0–7.3% in other age groups, *P* = 0.0001) and those with diarrhea (17.9% compared to 7.7% in those with no diarrhea, *P* = 0.006). In contrast, *G. duodenalis* was detected in 161/396 (40.7%), with infection rates of 48.3% (100/207), 35.9% (28/78), and 29.7% (33/111) in December 2011, June 2012, and September 2013, respectively. There were no significant gender- or diarrhea-associated differences, but the *G. duodenalis* infection rate in children of 13–24 months (50%) was significantly higher than in the age groups of 0–12 months and > 48 months (29.8–36.5%, *P* = 0.021). Co-infection of *Cryptosporidium* and *G. duodenalis* was seen in 19 (4.8%) children, but no *E. bieneusi* infection was detected in this study. All *Cryptosporidium*-positive specimens belonged to the subtype IaA14R4 of *C. hominis*, while all *G. duodenalis*-positive specimens belonged to sub-assemblage AII. Both were the same subtypes in a previous outbreak of cryptosporidiosis and giardiasis in a hospital ward hosting children from the welfare institute. Results of the study indicate that there was a persistent occurrence of limited *C. hominis* and *G. duodenalis* subtypes in the small enclosed community, with differences in age distribution and association with diarrhea occurrence between cryptosporidiosis and giardiasis.

## Introduction

*Cryptosporidium* spp., *Giardia duodenalis*, and *Enterocytozoon bieneusi* are common enteric pathogens in humans and animals, causing diarrhea in children, the elderly and AIDS patients. Humans become infected by these parasites through the fecal-oral route via direct contact with infected persons (anthroponotic route) or animals (zoonotic route) and ingestion of contaminated food or water (foodborne or waterborne route) (Xiao, 2010; Feng and Xiao, 2011; Santin and Fayer, 2011). Children are particularly vulnerable to infections, especially those who live in enclosed, crowded and insanitary environment such as daycare centers. In addition to diarrhea, cryptosporidiosis, giardiasis, and microsporidiosis in this population could cause malnutrition and cognitive impairments (Dillingham et al., 2002; Oliveira-Arbex et al., 2016).

Over 30 *Cryptosporidium* species and more than 40 genotypes have been identified to date, but *Cryptosporidium hominis* and *C. parvum* are responsible for > 90% of cryptosporidiosis cases in humans (Feng et al., 2018). Similarly, among the eight described assemblages (A–H) of *G. duodenalis* genotypes based on genetic characterizations, assemblages A and B are the causes of almost all cases of human giardiasis (Feng and Xiao, 2011). Multiple groups of genotypes with various host ranges are also present within *E. bieneusi*, with humans being mostly infected with Group 1 genotypes (Thellier and Breton, 2008). The identification of *Cryptosporidium* spp., *G. duodenalis*, and *E. bieneusi* at the species/genotype and subtype levels requires the use of molecular diagnostic tools. As different species and subtypes have different host ranges, the use of molecular diagnostic tools in epidemiological investigations has significantly improved our understanding of the transmission of *Cryptosporidium* spp., *G. duodenalis* and *E. bieneusi* in general populations (Thellier and Breton, 2008; Xiao and Feng, 2017).

Microscopy studies in Egypt, Peru, Thailand, Turkey, and some Eastern European countries have indicated a common occurrence of cryptosporidiosis, giardiasis and microsporidiosis in children in welfare institutes (Janoff et al., 1990; Makhlouf et al., 1994; Leelayoova et al., 2005; Borekci and Uzel, 2009; Bailey et al., 2013; Boontanom et al., 2014; Strkolcova et al., 2016; Kasprzak et al., 2017). However, the persistence of enteric pathogens in welfare institutes has not been examined longitudinally or using molecular diagnostic tools. In addition, there are no data on the occurrence of cryptosporidiosis, giardiasis and microsporidiosis in welfare institutes in China. Therefore, molecular tools were used in the present study to assess the occurrence, persistence and genetic characteristics of *Cryptosporidium* spp., *G. duodenalis*, and *E. bieneusi* in a welfare institute in Shanghai, China.

## Materials and Methods

### Ethics Statement

Fecal specimens in this study were collected as part of the public health investigation of a hospital-associated outbreak of cryptosporidiosis and giardiasis in inpatients from one welfare institute in Shanghai, China (Feng et al., 2012; Wang et al., 2013). During that investigation, children in one hospital ward hosting inpatients from the welfare institute had high occurrence of two subtypes of *C. hominis* and one subtype of *G. duodenalis* assemblage A over a 14-month period. Informed consent and permission for the collection of fecal specimens from diapers were obtained from the welfare institute manager and custodians of the children. The attending physician of the welfare institute submitted the fecal specimens to investigators of this study as part of a request to investigate the cryptosporidiosis and giardiasis outbreak. The research personnel had no physical access to the welfare institute and no direct contact with children in the welfare institute. Information provided by caregivers at the sampling included only age and gender of the children sampled and the presence or absence of diarrhea. As work in this report is part of the routine clinical diagnosis of pathogens for diarrhea in a health care facility as well as a public health investigation of an outbreak, the research protocol was considered an exempted human subject research. It was reviewed and approved by the Ethics Committee of East China University of Science and Technology.

### Specimen Collection

A total of 396 fresh fecal specimens were collected in December 2011 (207 specimens), June 2012 (78 specimens), and September 2013 (111 specimens) from children in the welfare institute in Shanghai, China. These children were 1–125 months in age, including 203 boys, 184 girls, and 9 with missing data. Among the specimens collected, 84 were from children with diarrhea. These children were mostly abandoned because of serious illnesses, including neurological/mental disability, growth retardation, cardiovascular diseases, and other conditions. Specimens were stored at 4°C in 2.5% potassium dichromate until detection of pathogens using molecular diagnostic tools.

### DNA Extraction

Each fecal specimen was washed twice with distilled water and centrifuged at 2,000 × g for 10 min to remove potassium dichromate. DNA was extracted from 200 μl of the washed fecal material using the FastDNA SPIN Kit for Soil (MP Biomedicals, Santa Ana, CA, United States), and stored at -80°C until being analyzed by PCR.

### PCR Analysis

A PCR-restriction fragment length polymorphism (RFLP) analysis of the small subunit (*ssu*) *rRNA* gene was used in the detection and genotyping of *Cryptosporidium* spp. (Xiao et al., 2001). To identify *Cryptosporidium* subtypes involved, a ∼850-bp fragment of the 60 kDa glycoprotein (*gp60*) gene was amplified by nested PCR (Feng et al., 2009). The *C. hominis* subtype identified was further characterized by a multilocus sequence typing (MLST) tool targeting genes encoding the 47 kDa protein (*cp47*), 56 kDa trans-membrane protein (*cp56*), serine repeat antigen (*msc6-7*), 70 kDa heat shock protein (*hsp70*), retinitis pigmentosa GT-Pase regulator (*rpgr*) and hydroxyproline-rich glycoprotein (*dz-hrgp*) (Gatei et al., 2006).

To detect *G. duodenalis*, a ∼530-bp fragment of the triosephosphate isomerase (*tpi*) gene was amplified by nested PCR (Feng et al., 2008). To identity multi-locus genotypes (MLGs) of *G. duodenalis*, a ∼511-bp fragment of the β-giardin (*bg*) gene (Caccio et al., 2008) and a ∼530-bp fragment of the glutamate dehydrogenase (*gdh*) gene (Abe et al., 2003) were amplified for all *tpi*-positive specimens, following the standard MLG terminology for *G. duodenalis* assemblage A (Feng and Xiao, 2011). For the detection of *E. bieneusi*, a nested PCR targeting a ∼530-bp fragment of the *rRNA* unit containing the entire 392-bp internal transcribed spacer (*its*) was used (Sulaiman et al., 2003).

In all PCR analyses, each specimen was analyzed twice using 1 μl of extracted DNA per PCR. Non-acetylated bovine serum albumin (Sigma-Aldrich, St. Louis, MO, United States) was used in the primary PCR at the concentration of 400 ng/μl to neutralize residual PCR inhibitors within the extracted DNA.

### Sequence Analysis

All positive secondary PCR products in this study were sequenced bi-directionally using the Big Dye Terminator v3.1 Cycle Sequencing Kits (Applied Biosystems, Foster City, CA, United States) on an ABI 3130 Genetic Analyzer (Applied Biosystems). Sequences were read and assembled using ChromasPro 1.5^1^ and compared with reference sequences in NCBI database using ClustalX^2^ to determine the genotypes and subtypes of pathogens. The novel *hsp70* gene sequence generated in this study was submitted to GenBank under the accession number MH329305.

### Data Analysis

The Chi-square test was used to compare differences in infection rates among samplings, age groups, genders and diarrhea status. Differences were considered significant at the level of *P* < 0.05. The statistical analysis was performed using the SPSS Statistics V21.0 for Windows (IBM Corp., New York, NY, United States). As children were sampled anonymously, no attempts were made to differentiate infection episodes among samplings.

## Results

### Occurrence of *Cryptosporidium* spp.

Among the 207 fecal specimens collected at the initial sampling in this study, 24 were positive for *Cryptosporidium* spp. by the *ssu rRNA*-based PCR, with an infection rate of 11.6% (Table 1). The infection rates were 24.6% (14/57), 11.9% (8/67), 3.4% (1/29), 0% (0/15), and 2.6% (1/39) in children of 0–12, 13–24, 25–36, 37–48, and > 48 months, respectively (*P* < 0.05 between 0–12-month and other age groups, except for the 13–24-month group, Table 2). The *Cryptosporidium* infection rate was about two-fold higher in children with diarrhea (19.6% or 9/46) than those without diarrhea (9.3% or 15/161), but the difference did not reach statistical significance (*P* = 0.056) (Table 2).

**Table 1:** Occurrence of *Cryptosporidium hominis* and *Giardia duodenalis* among 396 specimens from children in a welfare institute in Shanghai, China.

Sampling Time	No. of specimens	No. positive for *Cryptosporidium* (%)	Species/ subtype (No.)	No. positive for *G. duodenalis* (%)	Subtype at 3 genetic loci				MLG Type		
					*tpi*	*bg*	*gdh*		AII-1	AII-8	AII-13
December 2011	207	24 (11.6)	*C. hominis* (24)/IaA14R4 (15)	100 (48.3)	A2 (92)	A2 (47)/A3 (34)/A8 (7)	A2 (81)		42	30	4
June 2012	78	7 (9.0)	*C. hominis* (7)/IaA14R4 (7)	28 (35.9)	A2 (28)	A2 (21)	A2 (19)		18	–	–
September 2013	111	8 (7.2)	*C. hominis* (8)/IaA14R4 (4)	33 (29.7)	A2 (33)	A2 (25)/A3 (4)	A2 (28)		23	3	–
Total	396	39 (9.9)	*C. hominis* (39)/IaA14R4 (26)	161 (40.7)	A2 (153)	A2 (93)/A3 (38)/A8 (7)	A2 (128)		83	33	4

**Table 2:** Occurrence of *Cryptosporidium hominis*, *Giardia duodenalis*, and co-infections of the two among 396 specimens from children in a welfare institute in Shanghai, China by age, gender, diarrhea status, and sampling time.^∗^

Group	*Cryptosporidium hominis*					*Giardia duodenalis*					Co-infection
	1st sampling	2nd sampling	3rd sampling	Subtotal	*P-*value	1st sampling	2nd sampling	3rd sampling	Subtotal	*P-*value	
Age (month)					*P* = 0.0001					*P =* 0.081	
0–12	24.6 (14/57)	16.7 (7/42)	18.4 (7/38)	20.4 (28/137)		50.9 (29/57)	31 (13/42)	21.1 (8/38)	36.5 (50/137)		7.3 (10/137)
13–24	11.9 (8/67)	0 (0/23)	2.9 (1/34)	7.3 (9/124)		55.2 (37/67)	39.1 (9/23)	47.1 (16/34)	50 (62/124)		5.6 (7/124)
25–36	3.4 (1/29)	0 (0/9)	0 (0/21)	1.7 (1/59)		55.2 (16/29)	55.6 (5/9)	19.0 (4/21)	42.4 (25/59)		1.7 (1/59)
37–48	0 (0/15)	0 (0/1)	0 (0/13)	0 (0/29)		40 (6/15)	100 (1/1)	23.1 (3/13)	34.5 (10/29)		0 (0/29)
>48	2.6 (1/39)	0 (0/3)	0 (0/5)	2.1 (1/47)		30.8 (12/39)	0 (0/3)	40 (2/5)	29.8 (14/47)		2.1 (1/47)
Gender					*P =* 0.622					*P =* 0.237	
Male	13.5 (15/111)	5.4 (2/37)	3.6 (2/55)	9.4 (19/203)		50.5 (56/111)	35.1 (13/37)	32.7 (18/55)	42.9 (87/203)		4.9 (10/203)
Female	10.3 (9/87)	12.2 (5/41)	10.7 (6/56)	10.9 (20/184)		50.6 (44/87)	36.6 (15/41)	26.8 (15/56)	36.9 (68/184)		4.9 (9/184)
Unknown	0 (0/9)	0 (0/0)	0 (0/0)	0 (0/0)		66.7 (6/9)	0 (0/0)	0 (0/0)	6.7 (6/9)		0 (0/9)
Diarrhe					*P =* 0.006					*P =* 0.832	
Yes	19.6 (9/46)	17.6 (3/17)	14.3 (3/21)	17.9 (15/84)		56.5 (26/46)	11.8 (2/17)	33.3 (7/21)	41.7 (35/84)		10.7 (9/84)
No	9.3 (15/161)	6.6 (4/61)	5.6 (5/90)	7.7 (24/312)		46 (74/161)	42.6 (26/61)	28.9 (26/90)	40.4 (126/312)		3.2 (10/312)
Total	11.6 (24/207)	9 (7/78)	7.2 (8/111)	9.8 (39/396)		48.3 (100/207)	35.9 (28/78)	29.7 (33/111)	40.7 (161/396)		4.8 (19/396)

*^∗^Data are presented as percent of infection rate (No. of spositive/No. of specimens).*

Among the 78 and 111 specimens examined at the second and third sampling, 7 (9.0%) and 8 (7.2%) were positive for *Cryptosporidium* spp., respectively. The difference in *Cryptosporidium* infection rates was not significant among the three samplings (*P* = 0.438). As observed at the initial sampling, the age group of 0–12 months had higher infection rates at the second (16.7% or 7/42) and third (18.4% or 7/38) samplings than other age groups (0–2.9%). Likewise, children with diarrhea had a higher infection rate than those without diarrhea at the second (17.6% compared with 6.6%; *P* = 0.171) and third (14.3% compared with 5.6%; *P* = 0.173) sampling (Table 2).

Overall, *Cryptosporidium* spp. were detected in 39 (9.8%) of the 396 specimens examined in the study, with a higher infection rate in children of 0–12 months (20.4%) than in other age groups (0–7.3%) (Table 2). Although there was no significant difference in infection rates between boys (9.4% or 19/203) and girls (10.9% or 20/184), children with diarrhea had a significantly higher infection rate than those without diarrhea (17.9% or 15/84 compared with 7.7% or 24/312, *P* = 0.006).

### Occurrence of *G. duodenalis*

Among the 207 specimens collected at the initial sampling in this study, 100 (48.3%) were positive for *G. duodenalis* by the *tpi*-based PCR (Table 1). The infection rates were 50.9% (29/57), 55.2% (37/67), 55.2% (16/29), 40.0% (6/15), and 30.8% (12/39) in children of 0–12, 13–24, 25–36, 37–48, and > 48 months, respectively (*P* < 0.05 between > 48-month and other age groups, except for the 37–48-month group; Table 2). The *G. duodenalis* infection rate was higher in children with diarrhea (56.5% or 26/46) than in those without diarrhea (46% or 74/161), but the difference was not significant (*P* = 0.206) (Table 2).

Among the 78 and 111 specimens examined at the second and third sampling, 28 (35.9%) and 33 (29.7%) were positive for *G. duodenalis*, respectively. The difference in *G. duodenalis* infection rates was significant among the three sampling periods (*P* = 0.004). Different from the observation at the initial sampling, children in age groups of 25–36 months (55.6% or 5/9) and 13–24 months (47.1% or 16/34) had highest infection rate at the second and third sampling, respectively. Children with diarrhea had a slightly higher infection rate than those without diarrhea at the third sampling (33.3% compared with 28.9%, *P* = 0.792) but a lower infection rate at the second sampling (11.8% compared with 42.6%; *P* < 0.05; Table 2).

Overall, *G. duodenalis* was detected in 161 (40.7%) of the 396 specimens examined in the study, with a higher infection rate in children of 13–24 months (50%) than in other age groups (29.8–42.4%) (Table 2). There were no significant differences in infection rates between boys (42.9% or 87/203) and girls (36.9% or 68/184), or children with and without diarrhea (41.7% or 35/84 compared with 40.4% or 126/312, *P* = 0.832).

### Concurrence of *Cryptosporidium* spp. and *G. duodenalis* and Absence of *E. bieneusi*

Concurrent *Cryptosporidium* and *G. duodenalis* infection was detected in 4.8% (19/396) of the specimens analyzed (Table 2). The rate was similar between boy and girl (4.9% or 10/203 and 4.9% or 9/184, respectively). Children of 0–12 (7.3% or 10/137) and 13–24 (5.6% or 7/124) months were more likely to have co-infection of the two pathogens than other age groups (0–2.1%). Children with diarrhea (10.7% or 9/84) had a significantly higher concurrence of the two pathogens than those without diarrhea (3.2% or 10/312, *P* = 0.008).

None of the 396 fecal specimens analyzed in the study were positive for *E. bieneusi*.

### *Cryptosporidium* Species and Subtypes

All *Cryptosporidium*-positive secondary PCR products of the *ssu rRNA* gene in this study were successfully analyzed by RFLP, showing the presence of only *C. hominis* in the 39 PCR-positive specimens. Among them, 26 were successfully subtyped by PCR and sequence analysis of the *gp60* gene, leading to the identification of the only subtype IaA14R4 (GenBank accession number KC734574) (Table 1). Among the 26 *gp60*-positive specimens, 17, 21, 22, 22, 23, and 23 specimens were successfully subtyped by PCR and sequence analysis of the *hsp70*, *cp47*, *cp56*, *dz*-*hrgp*, *msc6-7*, and *rpgr* genes, respectively. Sequences obtained in the study were identical to each other at each genetic locus (GenBank accession numbers KC734581 for *msc6-7*, KC734584 for *rpgr*, KC734585 for *dz*-*hrgp*, KC734587 for *hsp70*, KC734590 for *cp47*, and KC734593 for *cp56*), except for the *hsp70* locus, which had one single nucleotide substitution of T to C (nucleotide position at 268) in one specimen (GenBank accession number MH329305).

### *Giardia duodenalis* Genotype and Subtypes

Among the 161 *G. duodenalis*-positive specimens, *tpi* products from 153 specimens were successfully sequenced, showing the presence of subtype A2 (GenBank accession number U57897) at all three samplings (Table 1). Among the 161 PCR-positive specimens at the *tpi* locus, 138 were positive in the *bg*-based PCR, and 128 were positive in the *gdh*-based PCR. All these PCR products were successfully sequenced, showing the presence of subtypes A2 (in 93 specimens), A3 (in 38 specimens), and A8 (in 7 specimens) at the *bg* locus (GenBank accession numbers AY072723, FJ971415, and JX898208, respectively) and A2 (in 128 specimens) at the *gdh* locus (GenBank accession number AY178737). At the *bg* locus, subtype A2 was found in 47, 21, and 25 specimens at the first, second, and third sampling, respectively; subtype A3 was detected in 34 and 4 specimens at the first and third sampling, respectively; whereas subtype A8 was only found in 7 specimens at the initial sampling. Altogether, sequence data were available at all three loci for 120 *G. duodenalis*-positive specimens, generating three MLGs of *G. duodenalis* in this study. All the three MLGs belonged to the sub-assemblage AII of assemblage A, with 83 specimens being positive for AII-1, 33 for AII-8 and 4 for AII-13 (Table 1).

## Discussion

A common occurrence of cryptosporidiosis and giardiasis was seen in children in the welfare institute in this study. The 9.8% overall infection rate of *Cryptosporidium* spp. is in concordance with observations in previous studies of cryptosporidiosis in welfare institutes in Thailand (Janoff et al., 1990; Jongwutiwes et al., 1990), but significantly higher than results obtained from other studies in Egypt and Turkey (Makhlouf et al., 1994; Borekci and Uzel, 2009; Doni et al., 2013). The infection rate was similar at the first (11.6%), second (9.0%), and third (7.2%) samplings, suggesting that there was a persistent occurrence of *C. hominis* in this welfare institute. The 40.7% overall infection rate of *G. duodenalis* was significantly higher than infection rates in previous studies in welfare institutes in Thailand, Egypt, Turkey and Peru (Janoff et al., 1990; Makhlouf et al., 1994; Borekci and Uzel, 2009; Turhan et al., 2009; Bailey et al., 2013; Doni et al., 2013). There was a gradual reduction in *G. duodenalis* infection rates during the study period (48.3, 35.9, and 29.7% at the first, second and third sampling, respectively). The latter, however, are still significantly higher than those reported in children in the general community (Natividad et al., 2008; Wang et al., 2013; Tellevik et al., 2015; Squire and Ryan, 2017). In contrast to the frequent detection of *E. bieneusi* in children in developing countries (Leelayoova et al., 2005; Wang et al., 2013; Lobo et al., 2014; Yang et al., 2014), none of the specimens analyzed in the present study was positive for this pathogen.

Results of genotyping and subtyping of *Cryptosporidium* spp. and *G. duodenalis* support the persistent nature of their occurrence in the welfare institute. In the present study, we identified the occurrence of only one *C. hominis* subtype IaA14R4 in all *Cryptosporidium*-positive specimens. MLST analysis of the *C. hominis* strains has further supported the homogeneous nature of the pathogen, with only one nucleotide substitution observed in one specimen at the *hsp70* locus. Similarly, only limited genetic heterogeneity was seen in the assemblage A of *G. duodenalis*. At the *tpi* and *gdh* loci, all positive specimens were identified as having the subtype A2, while some minor sequence differences were observed at the *bg* locus, with A2 as the dominant subtype. Thus, relatively homogeneous populations of *C. hominis* and *G. duodenalis* have been circulating among children in the welfare institute during the 22-month study period, although we cannot fully exclude the possibility of introduction of other *G. duodenalis* strains via new orphans. Different from the observation in the present study, several *Cryptosporidium* species and *G. duodenalis* assemblages were found in children from developing countries, including multiple subtypes of *C. hominis* and *C. parvum* and both assemblages A and B of *G. duodenalis* (Sanchez et al., 2017; Squire and Ryan, 2017; Naguib et al., 2018).

The IaA14R4 subtype was one of two *C. hominis* subtypes (the other one was IdA19) involved in a hospital-associated outbreak of cryptosporidiosis in Shanghai over a 14-month period (Feng et al., 2012). Ward A of this hospital was an inpatient ward for ill children from the welfare institute. Similarly, the *G. duodenalis* subtypes found in children of the welfare institute were also found in patients from this ward during the outbreak of cryptosporidiosis (Wang et al., 2013). In contrast, children in other wards of the hospital had assemblage B in addition to assemblage A (Wang et al., 2013). The detection of the same AII subtypes of *G. duodenalis* in the present studies supports the contribution of children from the welfare institute to the nosocomial infections of multiple enteric pathogens in Ward A. The lack of *E. bieneusi* detection and *C. hominis* IdA19 subtype in the present study suggests that introduction of pathogens from the welfare institute was not entirely responsible for the cryptosporidiosis outbreak. Previously, *E. bieneusi* was detected in 10.8% (8/74) of children in Ward A during the outbreak (Wang et al., 2013).

Children in the study had unique age-associated transmission patterns of *C. hominis* and *G. duodenalis*. *Cryptosporidium* infection rate was the highest in the age group of 0–12 months (20.4%). This is in contrast to the peak occurrence of cryptosporidiosis in children of 1–4 years in previous Chinese and African studies (Chen et al., 1992; Squire and Ryan, 2017; Ukwah et al., 2017). For example, in rural Jiangsu, China, *Cryptosporidium* infection rates were 3.4–3.63% in healthy children of 1–3 years, compared to 2.3% in children under 1 year (Chen et al., 1992), while in urban and suburban areas of Nigeria, the infection rates were 10.7 and 5.7% in diarrheic children of 1–4 years and under 1 year, respectively (Ukwah et al., 2017). Similarly, children of 13–24 months had the highest *G. duodenalis* infection rate, which appears to be different from observations in other studies in China, the Netherlands and Africa (Dib et al., 2008; Ismail et al., 2016; Pijnacker et al., 2016; Squire and Ryan, 2017). Therefore, the peak occurrence of cryptosporidiosis and giardiasis in the welfare institute is earlier in age than that seen in other studies. This is probably a reflection of complicating health conditions of children in the present study. The different age patterns between *C. hominis* and *G. duodenalis* infections could be due to some intrinsic biological differences between the two microorganisms.

The reason for the persistent occurrence of limited *C. hominis* and *G. duodenalis* subtypes in these children is unclear. Congregation of susceptible hosts, poor health, especially mental retardation in some children, and poor hand-wash of caregivers after diaper changes and before feeding could have all contributed to the fecal-oral transmission and persistent occurrence of *C. hominis* and *G. duodenalis* in this welfare institute. The role of each of the risk factors can only be assessed through thorough field investigations with extensive collection of epidemiological data.

## Conclusion

In conclusion, data of the study have revealed a persistent occurrence of *C. hominis* and *G. duodenalis* in children in the study welfare institute, as indicated by the presence of only one subtype of each parasite over an extended period of time. Proper training of caregivers and improved hygiene should be implemented to reduce the occurrence of cryptosporidiosis and giardiasis in the study facility and the spread of these diseases to the adoption families and the general community.

## Author Contributions

YF and LX designed the study. YW, NL, YG, LW, and RW performed the experiments. YW, LW, YF, and LX performed the statistical analysis, interpreted the results, and developed the draft manuscript. All authors contributed to manuscript revisions and approved the final version for publication.

## Conflict of Interest Statement

The authors declare that the research was conducted in the absence of any commercial or financial relationships that could be construed as a potential conflict of interest.

## Footnotes

1 http://technelysium.com.au/ChromasPro.html

2 http://clustal.org/
